# Using the Internet to Seek Information About Genetic and Rare Diseases: A Case Study Comparing Data From 2006 and 2011

**DOI:** 10.2196/resprot.2916

**Published:** 2014-02-24

**Authors:** Tamandra Morgan, Johanna Schmidt, Christy Haakonsen, Janine Lewis, Maria Della Rocca, Stephanie Morrison, Barbara Biesecker, Kimberly A Kaphingst

**Affiliations:** ^1^ICF InternationalRockville, MDUnited States; ^2^Children's National Medical CenterWashington, DCUnited States; ^3^The Harvey Institute for Human GeneticsBaltimore, MDUnited States; ^4^National Human Genome Research InstituteBethesda, MDUnited States; ^5^Washington University School of MedicineSt Louis, MOUnited States

**Keywords:** rare disease, genetic disease, patient education, information seeking, Internet use

## Abstract

**Background:**

The Genetic and Rare Disease Information Center (GARD) is a major provider of Web-based information on genetic and rare diseases. Little is known about the type of Web-based information individuals seek about genetic and rare diseases or their reasons for seeking.

**Objective:**

The objective of this paper is to describe the types of Web-based information sought about genetic and rare diseases and the reasons for seeking it from GARD by examining inquiries from 2006 and 2011.

**Methods:**

There were 278 English-language email and Web-based inquiries posed to GARD by lay individuals (ie, patients, parents, and relatives), which were randomly selected from inquiries in 2006 (n=68) and 2011 (n=210) and examined using content analysis.

**Results:**

Most often in both years, individuals sought basic disease information (51/68, 75.0% and 132/210, 62.8%; *P*=.067) and information about treatment (17/51, 33.3% and 62/132, 47.0%; *P*=.095). Specifically, inquirers requested information about their disease prognosis (6/51, 11.8% and 23/132, 17.4%; *P*=.347) and made requests for specialists (8/68, 11.8% and 31/210, 14.8%; *P*=.536). In both 2006 and 2011, a substantial subset of inquirers requested information related to undiagnosed symptoms, representing 16.2% (11/68) and 11.9% (25/210; *P*=.362) of inquiries, respectively. Inquirers were significantly more likely to have seen a health care provider before contacting GARD (99/210, 47.1% vs 20/68, 29.4%; *P*=.010) and to ask about clinical research studies in 2011 than in 2006 (24/210, 11.4% vs 2/68, 2.9%; *P*=.037). In the 2011 data set, the majority of the inquirers were women (201/210, 95.7%). In our 2006 sample, men were the majority source of inquiries (54/68, 79.4%).

**Conclusions:**

Findings from this study indicate that lay people contacting a genetic and rare disease information center most often seek information about disease prognosis, finding a specialist, and obtaining a diagnosis for symptoms. Unique characteristics of individuals searching the Internet for genetic and rare diseases information, includes a growing interest in participating in clinical research studies and a desire to supplement or better understand information discussed during a visit with a health care provider. These efforts represent advancements in patient self-advocacy.

## Introduction

Findings from a 2012 national survey of more than 3000 US adults by the Pew Research Center’s Internet and American Life Project revealed that 1 in 3 Americans have used Internet resources to better understand a medical condition [1]. In order to determine a potential diagnosis for themselves or another person, 35% of US adults have used Internet resources [1]. Other research has shown that the number of Web-based health information seekers is increasing. The results from multiple surveys have shown that, people turn to the Internet for disease-specific information [2-4], diagnosis of symptoms [5], and for help in determining whether to seek medical attention [6]. Collectively, Web-based health seekers tend to be younger, more educated, and more affluent than other health information seekers [7].

Although previous studies have examined Web-based searching for health information in general, limited information is available about the Web-based searching experience of people who are living with a rare or genetic disease. A 2001 study on use of the Internet for genetics-related information found that a majority of individuals surveyed searched the Internet for genetics-related information after visiting a genetics clinic. The top reasons cited for searching the Internet for genetics information, included to find information in layman’s terms, to get information about treatment, and to get information about genetic research [8].

A 2011 national study of Internet users by the Pew Internet and American Life Project found that the majority of those surveyed who had a rare disease said that they turned to other people with the same health condition for advice and support. Much of their interaction with fellow patients happened online because they did not live near people affected by the same conditions [9]. Prior research has demonstrated that obtaining information about a diagnosis and disease management can be challenging and frustrating for individuals with rare diseases. Rare diseases are defined as those with a prevalence of fewer than 200,000 affected individuals in the United States (approximately 1 in 1500 people) [10-15]. Accessing information is a challenge for individuals living with a rare disease, resulting in informational challenges for several reasons. Often, achieving a definitive diagnosis is delayed and requires significant effort and resources from patients, their families, and their health care providers; some patients’ conditions are never diagnosed [16]. Even once a diagnosis is made, it often leads to a cascade of questions whose answers may be limited by an uncertain prognosis, limited information, and lack of treatment options [10,11,17,18]. A 2013 study on the Internet user profile of Italian families with rare diseases found that at least 90% of responders searched the Internet for information on diagnosis, treatment, and finding a physician specialist for their child’s disease [19]. For many rare diseases, there are inadequate resources for patients, including few health care providers who have experience with their conditions. The lack of knowledge about rare diseases among health care providers leaves patients and their families to become as knowledgeable as possible on their condition. Patients and families may feel isolated and even desperate for any information that can help inform decisions about their clinical management or adaptation to their condition [10].

Previous research has indicated that individuals living with stigmatizing conditions, which is common to many genetic and rare diseases, may demonstrate greater use of Web-based health information seeking [9,20]. Unlike traditional Web-based health information seekers, individuals living with a genetic or rare disease may be seeking information about their health via the Internet because of the lack of any other available resources [9]. Therefore, the type of information and motivations for seeking Web-based health information may be influenced by the rarity of an individual’s condition. As a case example, this study sought to describe the type of information individuals contacting the Genetic and Rare Diseases (GARD) Information Center were seeking as well as their motivations for obtaining that information.

## Methods

### Data Source

The GARD Information Center, supported by the National Center for Advancing Translational Sciences, Office of Rare Diseases Research, and the National Human Genome Research Institute, was established to assist the general public, including patients, family members, and health care providers, in finding reliable and timely information about genetic and/or rare diseases. GARD information specialists are genetic counselors who provide information about genetic and rare diseases in English and Spanish via a toll-free hotline and personalized written responses to inquiries. GARD also provides access to its database of disease-specific information on the Internet. Many replies to inquiries are posted on the GARD website question and answer section and inquirers are directed to our website (and external websites for additional resources). Through this public resource, Internet users can search for information on more than 6000 genetic and/or rare diseases and pose questions directly to an information specialist. In 2006, 54% of all inquiries were received via email. In 2008 the Web-based form was added to the GARD website. In 2011, 7% of inquiries were received via email and 53% via the Web-based form. As of 2013, GARD information specialists have responded to over 40,000 inquiries from the public. On average, GARD currently receives 22 inquiries per day with 77% coming from within the United States and 13% from an international location (10% are not reported). Approximately one-third of all inquiries received are from patients or at-risk individuals.

### Inquiries

#### Data Collection

Among the approximately 34,000 inquiries in the GARD database, there were 2000 written inquiries from 2011, 98% of which were in English. The sample of inquiries were randomly obtained from the GARD dataset of inquiries from 2006 and 2011 through a computer-generated randomized software applied to a chronological list of annual inquiries. Our analysis, was conducted on a random sample of 250 English-language inquiries (10%) posed to GARD by lay individuals (ie, patients, spouses, relatives, friends, and parents/guardians) in 2011. We also analyzed a sample of 68 inquiries made in 2006 that were randomly drawn from inquires in 2006. To provide a focus for the qualitative analysis, we chose to only examine information-seeking by lay individuals without specialized knowledge. Inquiries from health care providers, social workers, and students were therefore excluded. We also excluded inquiries made by fax, letter, phone, or voicemail, as well as international inquiries and foreign-language inquiries. Finally, we excluded inquiries for which we could not determine the person’s reasons for contacting GARD (ie, the person asked a question but did not provide context). Inquiries that were complicated, confusing, or out-of-scope were also excluded.

The final sample yielded 278 inquiries, each of which contained the person’s verbatim initial inquiry, inquiry origin (domestic), type of contact (email and Web-based form), gender, date received at the information center, the specific condition for which they were inquiring, primary language (English), and their reason for inquiry. No other demographic information was collected.

#### Data Analysis and Coding Schema

All inquiries were de-identified and analyzed using thematic analysis through the QSR Nvivo software. Our aim was to identify the reasons for seeking information and the type of information sought by individuals contacting GARD. We were interested in finding broad themes with which to characterize information-seeking behavior of those with questions concerning genetic and rare diseases. We then identified similarities and differences in themes between inquiries from 2006 and 2011. To test the reliability of our coding, a second individual coded 20% of the inquiries from 2006 and 2011. Coding discrepancies were discussed systematically among the 2 coders and any differences were reconciled. Since the coding was quite literal, there were very few initial discrepancies in the coding.

## Results

### Data Sampling

A random sample of 278 English-language email and Web-based inquiries posed to GARD by lay individuals (ie, patients, parents, and relatives) in 2006 (n=68) and 2011 (n=210) was analyzed (Table 1). A majority of participants from both datasets self-identified as patients and female.

### Participant Demographics

Tables 1 and 2 highlight the characteristics of inquirers from 2006 and 2011. In both years, the inquirers were most often patients. There were more spouses/relatives and females in 2011 than in 2006 (*P*≤.05); there were no male inquirers in the 2011 data set.

**Table 1:** Inquirer user category.

User category	2006 (n=68), n (%)	2011 (n=210), n (%)
Friend	5 (7.4)	4 (1.9)
Parent/guardian	13 (19.1)	52 (24.8)
Patient	34 (50.0)	94 (44.7)
Spouse/relative	10 (14.7)	60 (28.6)
Not stated	6 (8.8)	0 (0.0)

**Table 2:** Inquirer gender.

Gender	2006 (n=68), n (%)	2011 (n=210), n (%)
Male	54 (79.4)	0 (0.0)
Female	14 (20.6)	201 (95.7)
Did not state	0 (0.0)	9 (4.3)

### Basic Disease Information

Most of those who inquired about a specific condition requested general disease information. The majority of individuals (51/68, 75.0% in 2006 and 132/210, 62.8% in 2011; *P*=.067) seemed to have very little information on a condition and were looking for any disease information that might be helpful to their understanding of the condition:

I recently was diagnosed with Pattern Dystrophy, but was not given any real information on this eye disease. Do you have a website I could go to and gather information. Anything at all you could tell me would be truly appreciated.

Individuals also requested more specific disease information related to obtaining a diagnosis, signs and symptoms, management and treatment, and prognosis.

### Diagnosis

In 2006 and 2011, 16% of inquirers (2006: 8/51, 15.7; 2011: 21/132, 15.9%; *P*=.970) requested information on obtaining a clinical diagnosis for their particular medical condition. Individuals wanted to know how diagnoses were made, including the clinical work-up or particular test, along with where to go to obtain a diagnosis for a suspected condition:

I would like information on this or how I can get a specific diagnosis. They ran a blood test but it came back non-specific. What would be the best way and the best kind of doctor to find out exactly what I have?

### Signs and Symptoms

About 7.8% (4/51) of inquirers in 2006 and 15.9% (21/132) of inquirers in 2011 requested information on the signs and symptoms of a condition (*P*=.154):

It is my understanding that hyperostosis frontalis interna can be a symptom of Morels syndrome. What are the symptoms of Morels syndrome?

### Management/Treatment

Of those who requested basic disease information, 33.3% (17/51) in 2006 and 47.0% (62/132) in 2011 (*P*=.095) requested information about management and treatment options, indicating that they found it difficult to find effective treatment options or providers who knew how to treat their condition:

I have a 15 year old daughter who was recently diagnosed with AAA syndrome... I would like to know if there is anything that can be done about her abnormal sweating she sweats profusely when she is cold it doesn’t matter how many layers of clothing she wears she sweats through them all. We have tried various types of treatments and so far none have worked. We welcome any ideas thank you for your time.

### Prognosis

Among those who wanted information about a specific condition, 11.8% (6/51) and 17.4% (23/132) of inquirers in 2006 and 2011, respectively, requested information on prognosis (*P*=.347). Frequently, inquirers wanted to know what issues they could expect to encounter when living with a particular condition:

I have a two year old who looks like he has this according to the genetic blood test done. A geneticist has not been assigned to his case yet and we lose our insurance coverage in 20 days. There is not a lot of information out there on what to expect in terms of how this can affect one’s life and was hoping for more information.

### Finding a Specialist

Many inquirers were looking for a specialist in their particular condition (8/68, 11.8% in 2006 and 31/210, 14.8% in 2011; *P*=.536). Some individuals reported having already seen a health care provider who was not particularly knowledgeable about their condition; therefore, they were hoping to find an expert:

She begged me to help her find someone that specializes to help her. I have looked but could not locate information as to where I might direct her for help. Can you supply the names of facilities or doctors in the Atlanta or Knoxville TN area that we could send her to for consultation?

### Undiagnosed Symptoms

In both 2006 and 2011, requests for information related to undiagnosed symptoms represented 16.2% (11/68) and 11.9% (25/210) of inquiries, respectively (*P*=.362). More specifically, inquirers with undiagnosed symptoms requested information about obtaining a specific diagnosis for their condition, getting the appropriate treatment, and finding a provider:

My daughter and I need a diagnosis for a rare disease we both have. We are both HLAB27. Both have duplicated urinary tracts, kidney both right sided. Both have cervical rib, severe joint pain, SI joint pain, migraines, carpel tunnel…and so much more. We need to find out the name of what we have. All rheumatologists we have seen are stumped plus other specialists. We are told over and over I have never seen this before- very strange.

### Previously Seen by a Health Care Provider

Several inquiries mentioned visiting a health care provider prior to contacting GARD. Many individuals reported seeing various doctors who were unable to either diagnose their condition or provide effective treatment. Inquirers from 2011 were statistically more likely to have seen a health care provider than inquirers from 2006 ((99/210, 47.1% vs 20/68, 29.4%, *P*=.010):

I am a 29 year old woman and I have symptoms of a rare illness for the past two years. I have visited several doctors and did numerous tests however the doctors were not able to determine what my illness is. I would like to know what this organization does and if you can be of any help to me. I would be grateful for any information. I am a 29 year old woman and I have symptoms of a rare illness for the past two years. I have visited several doctors and did numerous tests however the doctors were not able to determine what my illness is. I would like to know what this organization does and if you can be of any help to me. I would be grateful for any information.

### Research Studies

Often inquirers requested information about participating in research studies. In 2011, inquirers were significantly more likely to ask about clinical research trials as compared with those in 2006 (24/210, 11.4% vs 2/68, 2.9%; *P*=.037):

Asking if there is a clinical trial for treatment of oligodontia for children? I have five grandchildren and three of them are afflicted with a form of this malady but none of the three have the same symptoms.

## Discussion

### Principal Findings

The 2013 Pew research study found that half of Web-based health information searches are typically done on behalf of someone else [1]. In both 2006 and 2011, the most common type of GARD inquirers were those self-identified as patients. This novel finding may reveal the extent to which patients with genetic and rare diseases act as their own health care advocates. Patients in need of genetics-related information are often seeking more information after they have seen a health care provider [21], suggesting that health care providers frequently have limited resources and/or knowledge about many genetic and rare diseases. As a result, at least some patients may do their own research about their diagnosis or symptoms after visiting a provider. A study of Web-based health information seekers found that over 40% of Internet users had already sought help from a health professional for the same health issue prior to their Internet search [22]. Approximately 29% of inquiries in 2006 and 47% of inquiries in 2011 mentioned having already seen a health care provider prior to contacting GARD. The difference in inquiries may also reflect changes in health care delivery that suggest physicians have less time to spend with patients during clinic visits [23].

Inquirers in 2006 and 2011 were most often looking for information related to a specific disease or health concern. They also wanted to know about treatment and management options for a particular condition (33% in 2006 and 47% in 2011). The need for information on a specific condition and treatment options is a primary reason for Web-based health searches in more general samples. Previous research indicates that 66% of Internet users look for Web-based information about a specific disease or health concern and 56% look for information about medical treatment options [1]. The Web-based, information-seeking habits of parents of children with a rare health condition has been suggested to be rooted in an attempt to manage parental uncertainty [24]. Furthermore, prior findings in the literature on Web-based health information seeking for genetics-related information indicate that the prognosis for a condition and help finding a specialist is often desired [21]. Taken together, these categories are likely to be unique to the genetic- and rare-disease population because they reflect the difficulty patients have in finding information on their long-term outlook and providers with expertise in their particular condition.

Prior research on Internet searching for genetic information found that 46% of surveyed users visiting a genetics information website (ie, AsktheGeneticist) did so in order to find information about a possible diagnosis for themselves, their child, or other family relatives [21]. We showed that many inquiries in our 2006 and 2011 samples were related to finding a diagnosis for undiagnosed symptoms and conditions. The literature on “Web-based diagnosers” finds that 1 in 3 Americans have reported using the Internet to find a medical diagnosis for their symptoms and 1% of Web-based diagnosers say their follow-up conversation with a clinician regarding their suspected diagnosis was inconclusive [1]. Some of the inquirers dealing with undiagnosed symptoms in our sample may be Web-based diagnosers who have had inconclusive outcomes with their clinicians. Such inquiries were typically from individuals who had a long-standing medical condition for which they were unable to receive a diagnosis. These inquirers had often already seen several specialists and had extensive medical evaluations prior to requesting information from GARD. Taken together, the data suggest that existing resources, such as the Orphanet database are underused.

Between 2006 and 2011 there was a significant increase in the number of inquirers asking questions about participating in research studies. This finding may suggest an increase in people’s awareness of or interest in participation in research. The rising interest in clinical research participation among patients with genetic or rare diseases may have implications for health care providers in their decision to inform patients about these studies as well as explaining and facilitating the process of clinical research participation. Additionally, the need for more research in rare diseases is apparent in the numerous conditions that have no active clinical research studies available.

The results of this analysis have the potential to help improve GARD services. Knowing who is contacting GARD, what health-related information is of greatest interest, and how queries have changed between 2006 and 2011 will help GARD information specialists provide better, more targeted answers to future inquiries. The results of this study could also be shared with other groups to guide the development of targeted educational materials about genetic and rare diseases. For example, the increasing number of inquirers interested in research studies suggests a need for more information resources about participation in clinical research. Additionally, the number of individuals with undiagnosed diseases supports the need for more access to programs such as the National Institutes of Health Undiagnosed Diseases Program. Finally, these findings promote the relevance of the goals of the International Rare Disease Research Consortium for funding more research into better diagnostics and therapeutics for rare diseases.

### Limitations

 The limitations of this study pertain to the sampling design. First, data was sampled from two distinct time points, which limits our understanding of patients’ changing information needs over time. Second, the analysis was constrained by what was provided in an inquiry, without context about patients’ previous experiences. Additionally, since confusing or complicated inquiries were not analyzed as part of this study, our data may not include some of the reasons for seeking information from individuals with particularly challenging information needs and difficult situations. Third, in our qualitative analysis, a kappa statistic was not calculated. However, the independent coders concurred on the final coding. Fourth, because personal identifiers were not collected or were removed from the inquiries, we could not examine patterns based on many sociodemographic characteristics (ie, education level, income level, etc.) nor could we control for multiple inquiries coming from the same person. Fifth, in the 2011 data set, the majority of the inquirers were women. In our 2006 sample, men were the majority source of inquiries. This proportion arose from sampling bias because two-thirds of all GARD inquirers in 2006 were female, whereas only 18% were male and in 16% of cases, gender was not indicated. Randomization of the inquiries was key to the design in avoiding attention to specific types of inquires and deemed more important than drawing a sample representative of the sociodemographics.

### Conclusions

This study aimed to describe the types of information individuals contacting the GARD Information Center were seeking and the motivations behind obtaining this information. We determined that some types of information individuals contacting GARD requested was similar to those requested by other Web-based health information seekers. However, patients with genetic and rare diseases had unique types of queries regarding disease prognosis, identification of a disease specialist, and obtaining a diagnosis for undiagnosed symptoms and conditions. We also noted increased interest in participation in clinical research studies from 2006 to 2011. Further quantitative research is needed to examine predictors and outcomes of Web-based health information seeking among a larger population of patients with genetic and rare diseases.

## Abbreviations

GARD: Genetic and Rare Diseases
